# Microemulsion System for Topical Delivery of Thai Mango Seed Kernel Extract: Development, Physicochemical Characterisation and *Ex Vivo* Skin Permeation Studies

**DOI:** 10.3390/molecules191117107

**Published:** 2014-10-24

**Authors:** Jiraporn Leanpolchareanchai, Karine Padois, Françoise Falson, Rapepol Bavovada, Pimolpan Pithayanukul

**Affiliations:** 1 Department of Pharmacy, Faculty of Pharmacy, Mahidol University, 447 Sri-Ayuthaya Road, Rajathevi, Bangkok 10400, Thailand; E-Mail: jiraporn.lea@mahidol.ac.th; 2 EA 4169 “Fonctions Normales et Pathologiques de la Barrière Cutanée”, Laboratoire de Recherche et Développement de Pharmacie Galénique Industrielle, ISPB-Faculté de Pharmacie, Université Claude Bernard Lyon-I, 8 Avenue Rockefeller, F-69373 Lyon Cedex 08, France; E-Mails: poretk@hotmail.com (K.P.); francoise.falson@recherche.univ-lyon1.fr (F.F.); 3 Department of Pharmacognosy and Pharmaceutical Botany, Faculty of Pharmaceutical Sciences, Chulalongkorn University, 254 Payathai Road, Pathumwan, Bangkok 10330, Thailand; E-Mail: rapepol1@hotmail.com

**Keywords:** mango seed kernel extract, microemulsion, skin corrosion study, skin irritation study, skin permeation study, stability, topical delivery

## Abstract

A microemulsion system containing Thai mango seed kernel extract (MSKE, cultivar “Fahlun”) was developed and characterised for the purpose of topical skin delivery. The MSKE-loaded microemulsions were prepared by using the spontaneous emulsification method. Isopropyl myristate (IPM) was selected as the oil phase. A polyoxyethylene sorbitan monooleate and sorbitan monododecanoate (1:1, w/w) system was used as the surfactant phase; an aqueous mixture of different cosurfactants (absolute ethanol, 96.3% v/v ethanol, 1-propanol, 2-propanol or 1,2-propanediol) at a weight ratio of 1:1 was used as the aqueous phase. Among the cosurfactants studied, the 1-propanol aqueous mixture had the largest microemulsion region (48.93%) in the pseudo-ternary phase diagram. Microemulsions containing 1% MSKE demonstrated good physicochemical stability during a six-month study period at 25 ± 2 °C/60% ± 5% RH. The *ex vivo* skin permeation study demonstrated that the microemulsions exhibited a potent skin enhancement effect allowing MSKE to penetrate skin layers up to 60-fold higher compared with the control. Neither skin irritation nor skin corrosion was observed in *ex vivo* studies. The present study revealed that IPM-based microemulsion systems may be promising carriers to enhance skin penetration and delivering MSKE for topical treatment.

## 1. Introduction

Mango (*Mangifera indica* L.), which belongs to the family Anacardiaceae, is one of the most important fruits worldwide and is widely grown in over 100 countries in both tropical and subtropical regions, particularly in Asia. In 2011, Thailand, with 2.6 million tons produced, was the third largest mango producer in the world, following India (15.2 million tons) and China (4.4 million tons) [1]. The seed represents 10% to 25% of the whole fruit weight in different mango varieties [2]. The kernel inside the seed represents 45% to 75% of the seed and approximately 20% of the whole fruit [3]. More than one million tons of mango seeds are annually produced as wastes and are not currently utilised for any commercial purposes.

Phenolic compounds are widely found in the plant kingdom as secondary metabolites and have attracted considerable attention for their beneficial effects on human health [4]. Among the edible fruit portions, mango seed kernel extract (MSKE) has been reported to have a relatively high phenolic content and to exhibit potent antioxidant activity [5]. The ethanolic extract of Thai mango seed kernel cultivar “Fahlun” contains a relatively high phenolic content of pentagalloylglucopyranose (PGG) and relatively smaller amounts of methyl gallate and gallic acid [6]. MSKE and its principle components have been pharmacologically documented to have anti-methicillin-resistant *Staphylococcus aureus* and anti-tyrosinase properties, potent free radical scavenging, antioxidant, anti-inflammatory and hepatoprotective activities, as well as anti-enzymatic, anti-haemorrhagic and anti-dermonecrotic activities against snake venoms [6,7,8,9,10]. PGG possesses a broad spectrum of pharmacologic activities. With its multiple biological activities, it is a very promising novel drug candidate for the topical therapy of several diseases, including herpes simplex and allergic diseases [11,12].

The stratum corneum is the top layer of the skin, and it is difficult for most compounds to be delivered into and through it. A microemulsion is an isotropic, thermodynamically stable transparent or translucent system of oil, water and surfactant that is; frequently prepared in combination with a cosurfactant that has a 5–100 nm droplet size [13]. Due to their small droplet size, microemulsions have gained interest for pharmaceutical applications as carrier systems for dermal/transdermal drug delivery [14,15,16,17,18,19,20,21] because they provide several advantages over conventional topical formulations such as creams, ointments, and gels [18]. The objectives of this study were to develop and characterise microemulsion systems containing MSKE for the purpose of topical skin delivery. The MSKE-loaded microemulsions were prepared using the spontaneous emulsification method. Isopropyl myristate (IPM) was selected as the oil phase. A polyoxyethylene sorbitan monooleate (PSM) and sorbitan monododecanoate (SM) (1:1, w/w) system was used as the surfactant phase; an aqueous mixture of different cosurfactants (short-chain alcohol: absolute ethanol (EtOH), 96.3% v/v EtOH, 1-propanol (1-PA), 2-propanol (2-PA) or 1,2-propanediol (1,2-PA)) at a weight ratio of 1:1 was used as the aqueous phase. The physicochemical properties of the formulations were examined including conductivity, pH, refractive index, viscosity and rheological behaviour, and particle size. Their stabilities were evaluated under three different conditions for six months. Their skin permeation was determined using an *ex vivo* pig ear skin test. The skin irritation and corrosion effects of the formulations were also investigated using *ex vivo* tests.

## 2. Results and Discussion

### 2.1. Determination of Impurity Residues in MSKE

Because there are no specific limits for heavy metals, pesticide residues and microbial contamination in herbal extracts, the limits allowed for herbal raw materials and foods were used as guidance.

#### 2.1.1. Heavy Metal Residues in MSKE

The residues of heavy metals namely arsenic (As), cadmium (Cd), lead (Pb) and mercury (Hg) in MSKE were evaluated according to the Association of Official Analysis Chemists (AOAC). The contents of As, Cd and Hg could not be detected at the detection limits of 0.052, 0.020 and 0.004 ppm, respectively, while the content of Pb was less than the quantitative limit (<0.2 ppm). These results indicated that the heavy metal residues in MSKE were less than the allowed limits according to the Thai Herbal Pharmacopoeia 1995, Thai Food Acts 1986 and European Pharmacopoeia 2001.

#### 2.1.2. Pesticide Residues in MSKE

During economic plant cultivation and storage, agrochemicals and fumigants are usually used to control pests, particularly insects and fungi. Therefore, the pesticide residues (organophosphates, organochlorines, pyrethroids, and carbamates) in MSKE were determined according to the California Department of Food and Agriculture (CDFA) method. The results determined that pesticide residues could not be detected in MSKE.

#### 2.1.3. Microorganism Contamination in MSKE

The results determined that pathogenic bacteria; including *Salmonella* spp., *Clostridium* spp., and *Staphylococcus aureus* were not detected in MSKE.

### 2.2. MSKE Cytotoxicity

MSKE concentrations ranging between 1.56–200 µg/mL induced 1%–33% cell death in human dermal fibroblasts (HDF) cells. The extract concentration required to kill 50% (IC_50_) of cells was greater than 200 µg/mL. The results indicated that MSKE was not significantly cytotoxic as determined by greater than 50% cell viability at all tested doses.

### 2.3. MSKE Solubility

Solubility can be a useful indicator of how the extract will perform in microemulsion systems. MSKE solubility in the selected solvents was determined using the shake-flask method [22] and the equilibrium solubility values are shown in Table 1. The extract was most soluble in water and was more soluble in the aqueous mixture of short-chain alcohols (cosurfactant) than PSM, the surfactant mixture of PSM and SM (1:1, by weight), and SM, respectively. In addition, MSKE was insoluble in IPM. Recent findings suggested that MSKE should dissolve in an aqueous phase for microemulsion preparation [14,23].

**Table 1:** The equilibrium solubility of MSKE in the tested solvents at 25 ± 1 °C.

No.	Solvents	Equilibrium Solubility ^a,b^ (mg/g)
1.	IPM	0.00 ± 0.00
2.	Water	26.41 ± 1.60
3.	PSM	5.27 ± 1.83
4.	SM	0.02 ± 0.00
5.	Mixture of PSM and SM (1:1, by weight)	1.44 ± 0.70
6.	Absolute EtOH	1.81 ± 0.31
7.	96.3% EtOH	5.13 ± 0.98
8.	1-PA	0.69 ± 0.21
9.	2-PA	24.98 ± 1.50
10.	1,2-PA	13.22 ± 2.54
11.	Mixture of water and Absolute EtOH (1:1, by weight)	26.87 ± 1.16
12.	Mixture of water and 96.3% EtOH (1:1, by weight)	24.22 ± 2.79
13.	Mixture of water and 1-PA (1:1, by weight)	26.39 ± 2.32
14.	Mixture of water and 2-PA (1:1, by weight)	23.39 ± 1.19
15.	Mixture of water and 1,2-PA (1:1, by weight)	21.58 ± 4.18

IPM = isopropyl myristate, PSM = polyoxyethylene sorbitan monooleate, SM = sorbitan monododecanoate, EtOH = ethanol, 1-PA = 1-propanol, 2-PA = 2-propanol, 1,2-PA = 1,2-propanediol; ^a^ The values are expressed as the mean ± SD (*n* = 3); ^b^ The equilibrium solubility of MSKE in the tested solvents was calculated from the content analysis of PGG in MSKE.

### 2.4. MSKE Partition Coefficient

The MSKE partition coefficient was determined in IPM and water system. The measured log P_o/w_ was −1.65 ± 0.04 indicating that MSKE had a highly hydrophilic nature. Therefore, the extract should be restricted to the aqueous phase of the microemulsion formulation. Based on the partition coefficient and solubility data, it can be concluded that MSKE should have a high affinity for the hydrophilic component [24].

### 2.5. Pseudo-Ternary Phase Diagram

**Figure 1:**
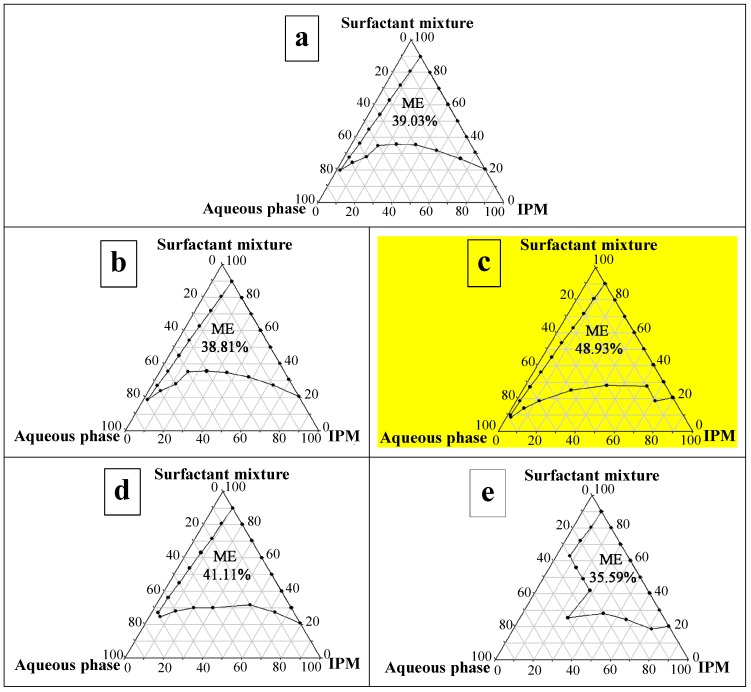
Pseudo-ternary phase diagrams of microemulsions composed of IPM, the surfactant mixture (PSM:SM = 1:1, by weight) and aqueous phase. The aqueous mixture contained different cosurfactants *i.e.*, short-chain alcohols: (**a**) absolute EtOH; (**b**) 96.3% EtOH; (**c**) 1-PA; (**d**) 2-PA; (**e**) 1,2-PA. The numbers shown in the areas represent the total area percentage of the microemulsion (ME) regions in the phase diagram.

The microemulsion components are acceptable for dermal use. IPM is used as an oil phase and a permeation enhancer in topical formulations [25]. The nonionic surfactants were selected to minimise the skin irritation and charge disruption of the system. The surfactants studied, PSM and SM, have previously been used in transdermal formulations [15,16]. According to previous studies [26,27], short-chain alcohol can decrease the hydrophilicity of the polar solvent and is capable of solubilizing high water content and promoting the microemulsion form; therefore, different types of short-chain alcohols (absolute EtOH, 96.3% EtOH, 1-PA, 2-PA, and 1,2-PA) were incorporated into the aqueous phase as a cosurfactant for phase diagrams in this study. Figure 1 demonstrates the transparent microemulsion region in the pseudo-ternary phase diagrams using the mixture of PSM and SM (1:1, by weight) as the nonionic surfactant pair, IPM as the oil phase, and the different types of short-chain alcohol as the cosurfactant.

The remaining region of the phase diagram represents turbid and conventional emulsions based on visual observation. As Figure 1 demonstrates using 1-PA as the cosurfactant provided the largest microemulsion area (48.93%). In addition, the formulations containing 1-PA and 2-PA as the cosurfactant had higher microemulsion areas than the formulations containing both ethanols as the cosurfactant. This result was in accordance with the results of El Maghraby [17] who reported that the increased length of short-chain alcohols from ethanol to isopropanol increased the microemulsion area. For formulations containing absolute EtOH and 96.3% EtOH as the cosurfactant, there was no significant difference between the total microemulsion area percentages in the phase diagram (*p* > 0.05).

### 2.6. Characterisation of Unloaded and MSKE-Loaded Microemulsions

All unloaded and MSKE-loaded microemulsions were clear yellowish liquids. They did not exhibit birefringence and appeared completely dark when observed under a cross-polarised light microscope, which indicated that they were optically isotropic. The visual appearance of microemulsions can be quite similar to lamellar and hexagonal liquid crystals. Cross-polarised light microscopy is the optimal method for differentiating liquid crystals. Under cross-polarised light microscopy, birefringence can be observed for lamellar and hexagonal liquid crystals but no birefringence is observed for microemulsions [28]. The formulation type was determined using a dilution test. As expected, the results determined that all water-in-oil (w/o) microemulsions could be diluted with IPM but not with brilliant blue aqueous solution. In contrast, all oil-in-water (o/w) microemulsions possessed the opposite properties. MSKE incorporation in the formulation did not change the microemulsion type of all unloaded microemulsion formulations. One exception was the unloaded o/w microemulsion with 1,2-PA as the cosurfactant. After extract incorporation in this formulation, the microemulsion type changed from o/w microemulsion to w/o microemulsion. This result was confirmed using the dilution test and electrical conductivity measurement (Table 2 and Table 3). The electrical conductivity values of all unloaded and MSKE-loaded microemulsion formulations were zero in the w/o microemulsions (Table 2 and Table 3). The o/w microemulsions had high conductivity values due to the conductive property of the aqueous external pseudophase. Extract incorporation did not affect the electrical conductivity of the unloaded microemulsions. The apparent pH values of unloaded microemulsions were between 6.92 and 7.40 (Table 2). MSKE incorporation affected the pH of each formulation. As observed in Table 3, extract incorporation significantly reduced the pH values of all unloaded microemulsions (*p ˂* 0.05) (Table 3). The refractive index values of all MSKE-loaded microemulsions were similar to their blank counterparts (Table 2 and Table 3). The refractive index values of o/w microemulsions were significantly lower than w/o microemulsions (*p ˂* 0.05) due to the lower refractive index of water as the extenal pseudophase (1.3325 ± 0.0000) compared with IPM as the external pseudophase (1.4340 ± 0.0000). The apparent viscosity data for all unloaded and MSKE-loaded microemulsions at a 1000 s^−1^ shear rate at 25 ± 1 °C are presented in Table 2 and Table 3. All samples exhibited Newtonian flow behaviour, as expected for microemulsions [29]. Extract incorporation significantly affected microemulsion viscosity (*p ˂* 0.05); however, it did not change the general flow behaviour. The average droplet sizes of unloaded microemulsions were between 8 ± 1 and 29 ± 0 nm (Table 2). MSKE incorporation affected the droplet size of each formulation. Except w/o microemulsions containing 1,2-PA as the cosurfactant, there were no significant differences between the average droplet sizes of unloaded w/o microemulsions and MSKE-loaded w/o microemulsions (*p ˃* 0.05). In o/w microemulsions, there were significant differences between the average droplet sizes of unloaded microemulsions and MSKE-loaded microemulsions (*p ˂* 0.05). The average droplet sizes of MSKE-loaded microemulsions were between 9 ± 0 and 70 ± 1 nm (Table 3). This result indicates that microemulsions were formed because their sizes range from 5 to 100 nm [13]. The surface areas of microemulsions are assumed to be high because their droplet size is small. Therefore, microemulsion droplets settle close to the skin, thereby providing a high concentration gradient and improved drug permeation from the formulation [21].

**Table 2:** Physical characterisations of all unloaded microemulsion formulations.

Formulations	σ ^a,b^ (μS/cm)	pH ^a,b^	RI ^a,b^	η at 1000 s^−1 a,b^ (mPas)	z-ave ^a,b^ (nm)	PDI ^a,b^
**Unloaded w/o microemulsions**						
1. Absolute EtOH	0(0)	7.31(0.00)	1.4364(0.0000)	38.51(0.13)	17(1)	0.46(0.08)
2. 96.3% EtOH	0(0)	7.28(0.00)	1.4353(0.0000)	40.45(0.27)	17(0)	0.41(0.01)
3. 1-PA	0(0)	7.32(0.00)	1.4362(0.0000)	36.54(0.14)	10(0)	0.48(0.06)
4. 2-PA	0(0)	7.40(0.00)	1.4376(0.0000)	38.02(0.12)	8(1)	0.46(0.10)
5. 1,2-PA	0(0)	7.14(0.00)	1.4385(0.0000)	79.56(0.88)	9(0)	0.42(0.10)
**Unloaded o/w microemulsions**						
1. Absolute EtOH	30(0)	7.07(0.00)	1.4237(0.0000)	35.17(0.04)	23(0)	0.20(0.00)
2. 96.3% EtOH	30(0)	6.98(0.00)	1.4248(0.0000)	37.51(0.11)	22(0)	0.22(0.00)
3. 1-PA	20(0)	6.99(0.00)	1.4261(0.0000)	31.92(0.08)	21(0)	0.30(0.01)
4. 2-PA	20(0)	7.20(0.00)	1.4263(0.0000)	35.07(0.17)	19(0)	0.27(0.00)
5. 1,2-PA	10(0)	6.92(0.00)	1.4335(0.0000)	118.18(0.07)	29(0)	0.29(0.01)

^a^ The mean of triplicate experiments; ^b^ The experiments were performed at 25 ± 1 °C; () = SD of triplicate experiments, σ = electrical conductivity, RI = refractive index, η = apparent viscosity, z-ave = mean particle size, PDI = polydispersity index.

### 2.7. Thermodynamic Stability of Unloaded and MSKE-Loaded Microemulsions

Microemulsions are thermodynamically stable systems and it is thermostability that differentiates microemulsions from emulsions that have kinetic stability and will eventually phase separate [18,30]. Consequently, the test formulations were subjected to different thermodynamic stability using centrifugation and freeze-thaw cycle stress tests. All unloaded and MSKE-loaded microemulsions exhibited good physical stability. No phase separation was observed. Only the unloaded o/w microemulsion containing 1,2-PA as the cosurfactant exhibited phase separation after the third cycle of the freeze-thaw stress test. This result indicated that this formulation was not thermodynamically stable. Only the formulations, that survived the thermodynamic stability tests were selected for the long term stability study [20,31].

**Table 3:** Physical characterisations of MSKE-loaded microemulsion formulations.

Formulations	σ ^a,b^ (μS/cm)	pH ^a,b^	RI ^a,b^	η at 1000 s^−1^ ^a,b^ (mPas)	z-ave ^a,b^ (nm)	PDI ^a,b^
**1% w/w MSKE-loaded w/o microemulsions**						
1. Absolute EtOH	0(0)	6.98(0.00)	1.4385(0.0000)	50.26(0.49)	16(0)	0.43(0.01)
2. 96.3% EtOH	0(0)	6.96(0.00)	1.4362(0.0000)	51.23(0.38)	17(0)	0.44(0.01)
3. 1-PA	0(0)	6.98(0.00)	1.4378(0.0000)	41.00(0.15)	10(0)	0.43(0.01)
4. 2-PA	0(0)	6.98(0.00)	1.4371(0.0000)	46.44(0.46)	9(0)	0.42(0.01)
5. 1,2-PA *	0(0)	6.84(0.00)	1.4385(0.0000)	97.87(0.58)	11(0)	0.42(0.02)
6. 1,2-PA **	0(0)	6.70(0.00)	1.4358(0.0000)	135.75(1.21)	70(1)	0.27(0.01)
**1% w/w MSKE-loaded o/w microemulsions**						
1. Absolute EtOH	30(0)	6.85(0.00)	1.4256(0.0000)	41.77(0.13)	21(0)	0.30(0.00)
2. 96.3% EtOH	30(0)	6.74(0.00)	1.4248(0.0000)	36.71(0.12)	33(0)	0.17(0.03)
3. 1-PA	20(0)	6.78(0.00)	1.4283(0.0000)	34.33(0.09)	25(0)	0.29(0.00)
4. 2-PA	20(0)	6.68(0.00)	1.4280(0.0000)	33.61(0.32)	36(0)	0.20(0.01)

^a^ The mean of triplicate experiments; ^b^ The experiments were performed at 25 ± 1 °C; () = SD of triplicate experiments, σ = electrical conductivity, RI = refractive index, η = apparent viscosity, z-ave = mean particle size, PDI = polydispersity index; * = MSKE-loaded w/o microemulsion containing IPM (44.55%) and aqueous phase of 1,2-PA (14.85%); ** = MSKE-loaded w/o microemulsion containing IPM (29.70%) and aqueous phase of 1,2-PA (29.70%).

### 2.8. Stability of Unloaded and MSKE-Loaded Microemulsions

All unloaded microemulsion formulations exhibited good physical stability during the six-month study period at 40 ± 2 °C/75% ± 5% RH. There were no significant differences between the values of conductivity, pH, refractive index, viscosity and mean droplet size at *t* = 0 and at *t* = 6 months (*p* > 0.05). In MSKE-loaded microemulsions, all formulations that were stored under various storage conditions for six months, *viz.*, 5 ± 3 °C, 25 ± 2 °C/60% ± 5% RH and 40 ± 2 °C/75% ± 5% RH (except MSKE-loaded o/w microemulsion containing 2-PA as a cosurfactant and stored at 40 ± 2 °C/75% ± 5% RH) had good physical stability. There were no significant differences between the values of conductivity, pH, refractive index, viscosity and mean droplet size at *t* = 0 and *t* = 6 months (*p* > 0.05). The MSKE-loaded o/w microemulsion containing 2-PA as the cosurfactant and stored at 40 ± 2 °C/75% ± 5% RH for six months demonstrated physical instability. This formulation changed its appearance from a clear yellowish liquid to a turbid brown-yellowish liquid and the change was confirmed by the mean particle size measurement, where the average droplet size increased from 36 ± 0 nm to 59 ± 1 nm. The chemical stability study results suggested that MSKE-loaded microemulsion formulations had good chemical stability during the six-month study period at 25 ± 2 °C/60% ± 5% RH because the percent of MSKE remaining in the formulations was greater than 85%.

### 2.9. Ex Vivo Skin Permeation Study

In the present study, an *ex vivo* skin permeation study was performed using Franz-type glass diffusion cells to compare MSKE-loaded microemulsion formulations with the MSKE solution. No MSKE permeation through the pig skin into the diffusion cell receiver chamber was detected. In contrast, MSKE penetration through the pig skin layers was observed. The MSKE contents in the epidermis and dermis are reported in Table 4 and the skin penetration values are expressed as MSKE amount per tissue weight. It was observed that MSKE in an aqueous solution accumulated in the epidermis and dermis. When MSKE was incorporated into the microemulsion formulations, tendency for higher accumulation in the epidermis was observed. Moreover, the extract amount in the dermis tended to increase. Additionally, a high SEM can be observed in Table 4. As reported by Wester and Maibach [32], high individual and regional variation exists for *in vivo* and *in vitro* percutaneous absorption in humans as well as animals. 

The total MSKE amount that penetrated into the skin layers from microemulsion formulations was significantly different from MSKE solution (*p* *˂* 0.05). The microemulsion had a potent enhancement effect allowing increased MSKE skin penetration by 7.7–59.9-fold compared with MSKE solution. MSKE incorporation into the microemulsions allowed the extract to penetrate the skin because of the large amount of surfactants in the formulation that facilitate MSKE passage. This effect is primarily caused by compromising the barrier function of skin, as demonstrated by increased TEWL (Table 5). For the microemulsion type, MSKE absorption in the skin layers for o/w microemulsions was higher than w/o microemulsions. These results were in accordance with those of Rozman *et al.* [19] who reported that the incorporation of vitamins in the outer phase of microemulsions resulted in greater absorption than when the vitamins were in the inner phase. Comparing different cosurfactants, 1-PA had the highest total MSKE amount penetrated into the skin layers, followed by 2-PA, 1,2-PA, absolute EtOH, and 96.3% EtOH. These results agreed with previous studies [33]. Kai *et al.* [33] determined that the increased length of short-chain alcohols from ethanol to propanol could enhance the flux of a polar, non-electrolyte penetrant (nicotinamide) across hairless mouse skin *in vitro*. In addition, Chantasart *et al.* [34] reported that branched-chain alkanols had lower enhancer potency than 1-alkanols with the same molecular formula; the potency decreased as the hydroxyl group moved from the terminal end towards the centre of the enhancer alkyl chain.

**Table 4:** *Ex vivo* skin permeation study.

Samples	MSKE Content ^a,c^ (μg/g)		
	Epidermis	Dermis	Total
1% w/w of MSKE solution	26.78 ± 3.39	13.77 ± 2.19	40.55 ± 1.23
**1% w/w MSKE-loaded w/o microemulsions**			
1. Absolute EtOH	225.42 ± 27.64	123.48 ± 6.18	348.90 ± 21.54 ^b^
2. 96.3% EtOH	161.33 ± 17.00	150.79 ± 15.13	312.12 ± 9.07 ^b^
3. 1-PA	1132.50 ± 36.23	687.97 ± 19.67	1820.47 ± 17.22 ^b^
4. 2-PA	1075.59 ± 185.97	671.00 ± 116.23	1746.58 ± 130.00 ^b^
5. 1,2-PA *	1076.27 ± 184.88	610.77 ± 102.78	1687.04 ± 84.07 ^b^
6. 1,2-PA **	637.38 ± 108.65	402.12 ± 66.55	1039.50 ± 49.24 ^b^
**1% w/w MSKE-loaded o/w microemulsions**			
1. Absolute EtOH	327.23 ± 53.04	573.07 ± 87.26	900.30 ± 34.30 ^b^
2. 96.3% EtOH	130.26 ± 19.11	613.38 ± 12.33	743.64 ± 15.53 ^b^
3. 1-PA	1070.11 ± 68.72	1358.90 ± 234.21	2429.01 ± 166.17 ^b^
4. 2-PA	800.69 ± 138.21	1518.43 ± 159.00	2319.12 ± 29.05 ^b^

^a^ The values are expressed as the mean ± SEM (*n* = 3); ^b^ Significant difference between total MSKE content of all MSKE-loaded microemulsions and MSKE solution (*p* < 0.05); ^c^ The amount of MSKE that penetrated into the skin layers was calculated from the content analysis of PGG in MSKE; * = MSKE-loaded w/o microemulsion containing IPM (44.55%) and aqueous phase of 1,2-PA (14.85%); ** = MSKE-loaded w/o microemulsion containing IPM (29.70%) and aqueous phase of 1,2-PA (29.70%).

**Table 5:** Skin irritancy and skin corrosion study results using pig ear skin.

Samples	ΔTEWL ^a,b^ (g/m^2^/h)	F ^a,c^
Water	0.9 ± 0.3	**−**	
20% w/v of SDS solution	24.6 ± 2.2 ^d^	**−**	
0.9% w/v of NaCl solution	**−**	0.00 ± 0.00	
37% v/v of HNO_3_ solution	**−**	**−**0.50 ± 0.03	
1% w/w of MSKE solution	1.5 ± 0.9 ^e^	4.82 ± 0.42	
**1% w/w MSKE-loaded w/o microemulsions**			
1. Absolute EtOH	3.2 ± 1.0 ^f^	4.12 ± 0.47 ^g^	
2. 96.3% EtOH	4.0 ± 2.0 ^f^	7.47 ± 0.27 ^g^	
3. 1-PA	3.1 ± 1.9 ^f^	11.32 ± 0.83 ^g^	
4. 2-PA	4.1 ± 2.4 ^f^	6.59 ± 0.21 ^g^	
5. 1,2-PA *	4.8 ± 0.8 ^f^	7.00 ± 1.07 ^g^	
6. 1,2-PA **	4.7 ± 1.8 ^f^	6.99 ± 1.05 ^g^	
**1% w/w MSKE-loaded o/w microemulsions**			
1. Absolute EtOH	5.1 ± 0.8 ^f^	8.05 ± 0.47 ^g^	
2. 96.3% EtOH	5.6 ± 2.0 ^f^	7.85 ± 0.35 ^g^	
3. 1-PA	5.4 ± 1.5 ^f^	6.56 ± 0.25 ^g^	
4. 2-PA	5.9 ± 1.6 ^f^	9.56 ± 0.96 ^g^	

^a^ The values are expressed as the mean ± SEM (*n* = 3); ^b^ ΔTEWL = the mean absolute increase of transepidermal water loss; ^c^ F = corrosive factor; ^d^ Significant difference between ΔTEWL of SDS solution and water (*p* < 0.05); ^e^ No significant difference between ΔTEWL of MSKE solution and water (*p* > 0.05); ^f^ No significant difference between ΔTEWL of all MSKE-loaded microemulsions and water (*p* > 0.05); ^g^ No significant difference of corrosive factor among MSKE-loaded microemulsion formulations (*p* > 0.05); * = MSKE-loaded w/o microemulsion containing IPM (44.55%) and aqueous phase of 1,2-PA (14.85%); ** = MSKE-loaded w/o microemulsion containing IPM (29.70%) and aqueous phase of 1,2-PA (29.70%).

### 2.10. The Non-Perfused Pig Ear Test for Skin Irritance

The non-perfused pig ear test is based on the determination of the absolute increase in transepidermal water loss (TEWL) from the skin surface, following the exposure of the pig ear to the test material, as the endpoint to distinguish between irritants and non-irritants. The results demonstrated that all MSKE-loaded microemulsion formulations were non-irritants (ΔTEWL ˂ 6 g/m^2^/h) (Table 5). These results were in accordance with those of Fentem *et al.* [35] and Zuang *et al.* [36]. If the tested material induced an absolute increase in TEWL ≥ 6 g/m^2^/h, it was classified as an irritant. A statistical analysis confirmed that there was no significant difference between the absolute increase in TEWL for all MSKE-loaded microemulsions and water (*p* > 0.05).

### 2.11. Ex Vivo Skin Corrosion Study

Any application of substances onto the skin must not damage human health when applied under normal conditions. Therefore, any compound considered for human skin application must be tested for irreversible skin damage (skin corrosion). The corrosion study results demonstrated that all MSKE-loaded microemulsion formulations were non-corrosive (Table 5). These results coincide with those of Padois *et al.* [37], who reported that a solid lipid nanoparticle suspension containing 5% minoxidil was non-corrosive (F ≥ 0) while various tested commercial products exhibited a corrosive potential (F ˂ 0). A statistical analysis confirmed that there were no significant corrosive differences between the studied of microemulsion formulations (*p* > 0.05).

## 3. Experimental Section 

### 3.1. Materials

Pentagalloylglucopyranose (PGG; >95%) was obtained from Endotherm GmbH (Saarbrücken, Germany). Coomassie brilliant blue R-250 and sodium dodecyl sulphate (SDS) powder were purchased from Bio-Rad Laboratories (Hercules, CA, USA). Phosphate buffered saline (PBS) tablets used in the preparation of pH 7.4 buffer solution, 1-propanol (1-PA; ≥99.5%) and sulforhodamine B (SRB, dye content 75%) were obtained from Sigma Chemical Company (St. Louis, MO, USA). Sodium chloride (NaCl, AR grade) was purchased from Univar Australia Pty. Ltd. (Ingleburn, NSW, Australia). Polyoxyethylene sorbitan monooleate (PSM) and sorbitan monododecanoate (SM) were obtained from Seppic S.A. (Castres, France). Isopropyl myristate (IPM; ≥98%) and 2-propanol (2-PA; 99.7%) were purchased from Merck KGaA (Darmstadt, Germany). Absolute ethanol BP (99.6%) and ethanol BP (EtOH; 96.3%) were obtained locally from V.S. Technologies and Hexalab (Lyon, France), respectively. 1,2-Propanediol (1,2-PA; ≥99.5%) was obtained from VWR International Ltd. (Val-de-Marne, France). Acetonitrile (ACN; 99.9%; HPLC grade) was purchased from Panreac Química S.A.U. (Barcelona, Spain). Orthophosphoric acid (H_3_PO_4_; Normapur^TM^) was obtained from Prolabo (Paris, France). *n*-Octanol and nitric acid (HNO_3_) were purchased from Carlo Erba Réactifs-SdS (Val-de-Reuil, France). Ultrapure water by Ultralab_S (Colomiers, France) was used throughout the experiments. Other chemicals and reagents (analytical grade or higher) were obtained from local distributors and used as received without further purification.

### 3.2. MSKE Preparation

Fully grown unripened Thai mango fruits (*Mangifera indica* L. cultivar “Fahlun”) were purchased from a local market (Mahanak Market, Bangkok, Thailand). A voucher specimen (R.B. 20007) was deposited in the Museum of Natural Medicine, Faculty of Pharmaceutical Sciences, Chulalongkorn University, Bangkok, Thailand. The ethanolic extract from seed kernels was obtained using a previously described method [7]. Briefly, the chopped kernels (1.93 kg) were homogenised in hot ethanol (80 °C) and defatted with hexane. After the solvent was evaporated under reduced pressure, the remaining aqueous portion was freeze-dried to produce crude MSKE with a 9.36% (w/w) yield. The extract was stored in a vacuum desiccator at 4 °C until use.

**Figure 2:**
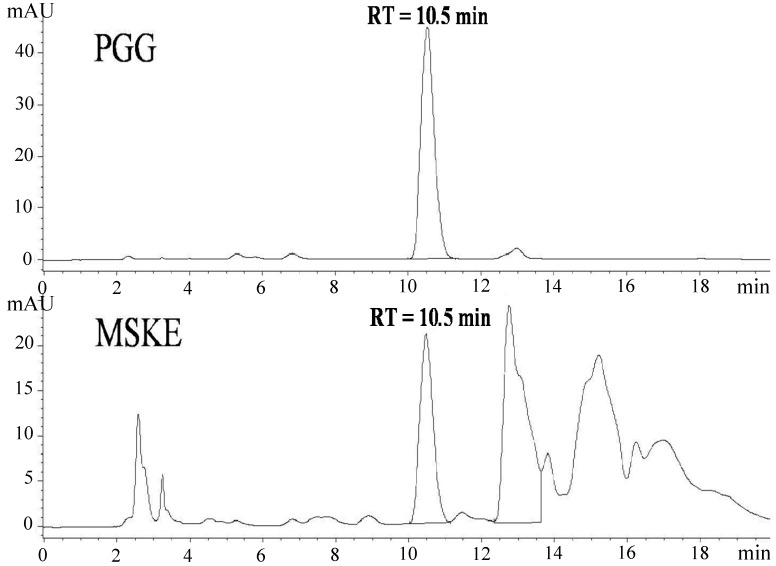
RP-HPLC chromatograms of the reference standard (PGG) and MSKE using a Gemini-NX C-18 reversed phase column (250 mm × 4.6 mm, 5 μm particle size) with a guard column. Mobile phase consisted of H_3_PO_4_ in water at pH 2 (solvent A) and ACN (solvent B). The total run time was 20 min and the gradient program was as follows: 20% B for 0–5 min, 20% B to 30% B for 10 min and 30% B to 20% B for 5 min. The flow rate was 1.1 mL/min. The injection volume was 75 μL. The UV detector was set at 280 nm.

### 3.3. Analysis of PGG Content in MSKE

The quantitative determination of PGG content in MSKE was performed using high performance liquid chromatography (HPLC) on an Agilent 1200 Series HPLC system (Agilent Technologies, Massy, France) equipped with a quaternary pump, a multiple wavelength detector, a vacuum degasser and an autosampler. The data were recorded and analysed using the Agilent ChemStation Rev. B.02.01 program. A Gemini-NX column (C18; 250 mm × 4.6 mm i.d., particle size 5 μm; Phenomenex Inc., Torrance, CA, USA) with a guard column was used as a stationary phase. Elution was performed using gradient solvent systems with a flow rate of 1.1 mL/min at room temperature (25 ± 1 °C). The mobile phase consisted of H_3_PO_4_ in water at pH 2 (solvent A) and ACN (solvent B). The total run time was 20 min and the gradient program was set as follows: 20% B for 0–5 min, 20% B to 30% B for 10 min, and 30% B to 20% B for 5 min. The samples (MSKE and the reference standard, PGG) were dissolved in ACN and the sample injection volume was 75 μL. UV detection was performed by monitoring the absorbance signal at 280 nm. The identification of PGG in MSKE was based on the comparison of the retention time and UV spectrum of the unknown sample peaks with the reference standard, PGG (Figure 2). The retention time of PGG was 10.5 min. The HPLC method was validated for its linearity, precision, accuracy, limit of detection (LOD) and limit of quantitation (LOQ) as per the International Conference on Harmonization (ICH) guidelines. The PGG calibration curve concentration range was 0.5025–10 μg/mL. The calibration curve linear equation was y = 114.67x − 4.0454 and the linear correlation coefficient was 1. The intra-day and inter-day precision of PGG was evaluated using three concentrations (0.5, 5 and 10 μg/mL) and the coefficient of variation was lower than 2%. The method accuracy was determined using a recovery study conducted at three different concentrations (0.8, 4 and 8 μg/mL), and the average recovery was 98.36 ± 1.48%. The LOQ and LOD for PGG chromatographic determination were 0.29 and 0.09 μg/mL, respectively. The amount of PGG present in MSKE was 9.24% ± 0.20% w/w.

### 3.4. Determination of Impurity Residues in MSKE

#### 3.4.1. Heavy Metal Residues Analysis

Heavy metal residues in MSKE namely, arsenic (As), cadmium (Cd), lead (Pb) and mercury (Hg) were determined according to the AOAC official method of analysis. The amounts of As and Hg were determined using flame atomic absorption spectroscopy [38], while the quantities of Cd and Pb were determined using graphite furnace atomic absorption spectroscopy [39]. The analyses were performed by the National Food Institute (NFI), Bangkok, Thailand.

#### 3.4.2. Pesticide Residues Analysis

Pesticide residues (organochlorines, organophosphates, pyrethroids and carbamates) in MSKE were determined using a multipesticide residue screening according to CDFA methods [40], developed by CDFA. The analyses were performed by NFI, Bangkok, Thailand.

#### 3.4.3. Microorganism Contamination Analysis

Selected pathogenic bacteria, *i.e.*, *Salmonella* spp., *Clostridium* spp. and *Staphylococcus aureus* were identified according to the method described in the United States Pharmacopeia 26. The analyses were performed by the Department of Microbiology, Faculty of Pharmacy, Mahidol University, Bangkok, Thailand.

### 3.5. MSKE Cytotoxicity Test

MSKE toxicity was assessed using the MTT (3-(4,5-dimethylthiazol-2-yl)-2,5-diphenyltetrazolium bromide) assay [41] using human dermal fibroblasts (HDF) obtained from normal foreskin biopsy. The assay was performed by Bioassay Laboratory, National Centre for Genetic Engineering and Biotechnology, Pathumthani, Thailand.

### 3.6. MSKE Solubility Determination

The solubility of MSKE was determined in the selected solvents. An excess amount of MSKE was added to 2 g of each selected solvent system. The sample mixtures were mechanically agitated using a shaking water bath operating at 100 strokes per min (spm) for 72 h at 25 ± 1 °C to reach equilibrium. Then, the equilibrated samples were centrifuged at 14,500 rpm for 10 min. The clear supernatants were obtained and diluted with the appropriate solvents. Next, the samples were filtered through a polytetrafluoroethylene (PTFE) membrane filter (φ = 0.45 µm) and the filtrates were assayed using HPLC. The solubility measurements were performed in triplicate and the equilibrium solubility of MSKE in the test solvents was calculated from the content analysis of PGG in MSKE.

### 3.7. MSKE Partition Coefficient Determination

The MSKE partition coefficient study was performed using the shake-flask method. IPM and water were used as the organic and aqueous phases, respectively. The two phases were mutually saturated before the experiment by mixing them in a 1:1 ratio (v/v) and shaking overnight on a shaking water bath operating at 100 spm for 24 h at 25 ± 1 °C. Then, the two phases were separated using a separating funnel. Further, 1 g of MSKE was added to the mixture of pre-saturated organic phase (10 mL) and pre-saturated aqueous phase (10 mL) in a 25-mL glass-stoppered conical flask. The flask was shaken for 24 h at 25 ± 1 °C in a shaking water bath. The two phases were then separated using a separating funnel and the MSKE concentration in each phase was determined using HPLC after appropriate dilution. The experiment was performed in triplicate. The MSKE partition coefficient was calculated from the content analysis of PGG in MSKE and used the following equation:

(1) $${\text{P}}_{\text{o/w}}\text{ = }\frac{{\text{C}}_{\text{o}}}{{\text{C}}_{\text{w}}}$$

where, P_o/w_ denotes the organic-aqueous partition coefficient. C_o_ and C_w_ are the equilibrium concentrations of MSKE in organic and aqueous phases, respectively.

### 3.8. Pseudo-Ternary Phase Diagram Construction

Pseudo-ternary phase diagrams of unloaded microemulsions were prepared. IPM was used as the oil phase and the system of PSM and SM at a weight ratio of 1:1 was used as the surfactant phase. The aqueous mixture of different cosurfactants (absolute EtOH, 96.3% EtOH, 1-PA, 2-PA, or 1,2-PA) at a weight ratio of 1:1 was used as the aqueous phase. For each phase diagram, the surfactant mixture was added into the oil phase, resulting in surfactant mixture:IPM weight ratios of 9:1, 8:2, 7:3, 6:4, 5:5, 4:6, 3:7, 2:8 and 1:9. These mixtures were vigorously mixed using a magnetic stirrer until a homogeneous dispersion was obtained. Afterwards, the aqueous phase was added, resulting in aqueous phase concentrations ranging from 0% to 90% at 10% weight intervals. The systems were stirred using a magnetic stirrer for 5 min and stored to reach equilibrium at 25 ± 1 °C for 24 h before further experimentation. The obtained formulations were defined as microemulsions with transparency character. The appearance of turbidity was considered an indication of phase separation. Visual observations were performed to identify microemulsions, gels or two-phase regions. Subsequently, the microemulsion type was determined through dilution tests with brilliant blue aqueous solution or IPM. The w/o microemulsions were expected to be immiscible in the brilliant blue aqueous solution, but miscible with IPM. The o/w microemulsions were expected to provide the opposite results. The microemulsion region of the system was constructed on a triangular graph using the SigmaPlot^®^ 10.0 software. A cut-and-weigh method was used to determine the total area percentage of the phase diagram covered by the microemulsions [42]. No attempts were undertaken to identify the regions of other association structures.

### 3.9. Preparation of Unloaded and MSKE-Loaded Microemulsions

After the microemulsion region in the phase diagram was obtained, formulations were selected at a 40% w/w surfactant mixture concentration which was likely to produce both o/w and w/o microemulsions. For o/w microemulsions, the IPM concentration was 30% w/w and the aqueous phase concentration was 30% w/w. The concentrations of IPM and aqueous phase were 45% and 15% w/w, respectively for w/o microemulsions. The extract was incorporated at a 1% w/w concentration in the microemulsion formulation, and the MSKE-loaded microemulsions were prepared according to the typical formulations as presented in Table 6. The MSKE-loaded microemulsions were prepared by incorporating MSKE in the aqueous phase, adding the mixture of PSM and SM, and mixing with the oil. All components were mixed together using a magnetic stirrer until a uniform mixture was obtained. The systems were stored to reach equilibrium at 25 ± 1 °C for 24 h before further experimentation. The obtained microemulsions were then characterised and compared with their blank counterparts.

**Table 6:** Compositions of unloaded and MSKE-loaded microemulsions.

Component	Unloaded Microemulsions		MSKE-Loaded Microemulsions	
	w/o (% w/w)	o/w (% w/w)	w/o (% w/w)	o/w (% w/w)
MSKE	-	-	1.00	1.00
PSM:SM (1:1, by weight)	40	40	39.60	39.60
IPM	45	30	44.55	29.70
Water:Cosurfactant ^a^ (1:1, by weight)	15	30	14.85	29.70

PSM = polyoxyethylene sorbitan monooleate; SM = sorbitan monododecanoate; ^a^ Cosurfactants were absolute EtOH, 96.3% EtOH, 1-PA, 2-PA and 1,2-PA.

### 3.10. Characterisation of Unloaded and MSKE-Loaded Microemulsions

#### 3.10.1. Macroscopic Observation

All samples were visually observed for their physical appearance, *i.e.*, clearness, colour and homogeneity. To verify the isotropic nature of the microemulsions, samples were examined using cross-polarised light microscopy (Eclipse E200 Microscope, Nikon Corporation Instruments Company, Tokyo, Japan) at a magnification of 10 × 100. A sample drop was placed between a cover slip and a glass slide and observed under cross-polarised light. The experiments were performed at 25 ± 1 °C.

#### 3.10.2. Conductivity Measurement

The sample electrical conductivity values were measured using a conductivity tester (ECTestr™ 11, Eutech Instruments Pte. Ltd., Singapore) and compared with the conductivity values of IPM or water alone to determine whether the microemulsions were oil-continuous or aqueous-continuous. The measurements were performed in triplicate at 25 ± 1 °C.

#### 3.10.3. pH Measurement

The apparent pH values of the samples were measured using a pH-meter (CyberScan pH110, Eutech Instruments Pte. Ltd.). The measurements were performed in triplicate at 25 ± 1 °C.

#### 3.10.4. Refractive Index Measurement

The refractive indices of the samples were determined using a digital hand-held pocket refractometer PAL-2 (Atago Co., Ltd., Tokyo, Japan). The measurements were performed in triplicate at 25 ± 1 °C.

#### 3.10.5. Viscosity and Rheological Behaviour Determination

The sample viscosity and rheological behavior were measured at different shear rates using a rotational viscometer with a cylindrical measurement system of stainless steel, comprising a measuring bob 1 (φ = 30 mm, l = 45 mm), measuring tube 1 (φ = 32.54 mm) and cap 1 (Rheomat RM180, Rheometric Scientific GmbH, Munich, Germany). The Rheomatic-P software was used to drive and calculate rheological data measurements from the viscometer. The MS DIN/ISO thermostating unit was connected to a circulating water bath operating at 25 ± 1 °C. A 30 mL sample volume was used for viscosity measurements. The rheological behaviour of the systems was examined by constructing a rheogram of shear stress against shear rate. The apparent viscosity data at the shear rate of 1000 s^−1^ and 25 ± 1 °C were obtained from the measurements. The experiments were performed in triplicate.

#### 3.10.6. Particle Size Measurement

To obtain accurate droplet size measurements, the refractive indices of the dispersed and continuous phases of the microemulsions were measured using a digital hand-held pocket refractometer PAL-1 (0%–53% Brix) and PAL-2 (45%–93% Brix). The viscosity of the continuous phase of the microemulsions was measured using a Rheomat RM180 viscometer. The refractive index and the viscosity values were employed during average droplet size analysis. The samples were then analysed in a Zetasizer Nano-ZS apparatus (Malvern Instruments Ltd., Worcestershire, UK) to calculate the average droplet size (z-ave) and polydispersity index (PDI) of the formulations. The measurement was obtained at a 173° detection angle and the measurement position within the cuvette was automatically determined by the software. The sample droplet size was calculated using the Stokes-Einstein relationship by the Zetasizer Software. Samples were not diluted before the measurement. The experiments were performed in triplicate at 25 ± 1 °C.

### 3.11. Thermodynamic Stability of Unloaded and MSKE-Loaded Microemulsions

To assess the thermodynamic stability of the formulations, the following two tests were performed.

#### 3.11.1. Centrifuge Stress Test

The samples were tested by centrifugation (Sigma 3-16P, SIGMA Laborzentrifugen GmbH, Osterode am Harz, Germany) at 14,500 rpm for 1 h at 25 ± 1 °C. After centrifugation, the physical instability of the formulations was observed which was visually determined by the degree of phase separation. The formulations that did not display phase separation were considered for the freeze-thaw stress test.

#### 3.11.2. Freeze-Thaw Stress Test

The formulations were submitted to a total of three complete cycles, each cycle consisting of 24 h at −20 °C followed by 24 h at 25 ± 1 °C. After the third cycle, the formulations were examined for their physical instability which was visually determined by the degree of phase separation. 

### 3.12. Stability Study of Unloaded and MSKE-Loaded Microemulsions

The physical stability of the unloaded microemulsion formulations was evaluated by monitoring the time-dependent change of the physical characteristics. The formulations were stored at 40 ± 2 °C/75% ± 5% RH as per the ICH guidelines for six months. For MSKE-loaded microemulsion formulations, the physical and chemical stability of the formulations were evaluated by monitoring time- and temperature-dependent changes of the physicochemical characteristics. The formulations were stored under various storage conditions, *viz.*, 5 ± 3 °C, 25 ± 2 °C/60% ± 5% RH, and 40 ± 2 °C/75% ± 5% RH as per the ICH guidelines, for six months. At predetermined times (immediately after preparation and at 1, 2, 3, 4, 5 and 6 months); all unloaded and MSKE-loaded microemulsion formulations were collected to evaluate their physical appearance, electrical conductivity, pH, refractive index, viscosity and rheological behaviour, and particle size. The chemical stability of MSKE in the formulations was analysed using HPLC and the amount of MSKE was calculated from the content analysis of PGG in MSKE. All measurements were performed in triplicate at 25 ± 1 °C.

### 3.13. Ex Vivo Skin Permeation Study

Full-thickness skin from the pig ear is an accepted permeation model for human dermatological research. Pig ears were obtained from a local slaughterhouse. They were washed using tap water and full-thickness skin was carefully removed from the dorsal side of the pig ear using a scalpel. Subsequently, the subcutaneous fatty tissue was carefully removed and the excised skin was immediately stored flat at −20 ± 1 °C until use. The *ex vivo* skin permeation study was performed using vertical Franz-type glass diffusion cells containing full-thickness pig skin. The pig skin was mounted in the two-chamber glass diffusion cell. The effective diffusion area was 0.785 cm^2^ and the receptor compartment contained 9 mL 0.01 M phosphate buffered saline (PBS; pH 7.4). The receptor compartment was continuously homogenised using a magnetic stir bar. The cells were placed in a temperature-controlled water bath at 37 ± 1 °C providing a skin surface temperature of 32 ± 1 °C. The skin was equilibrated with the receptor phase overnight and 0.5 g of each microemulsion formulation was spread uniformly on the stratum corneum side of the skin surface in the donor compartment. The MSKE solution (1% w/w; 0.5 g) was also applied in parallel. The MSKE solubility in PBS was determined to be 17.59 ± 2.02 mg/mL. Therefore, sink conditions were maintained throughout the experiment. The receptor phase (0.5 mL) was withdrawn at 0, 1, 2, 4, 6, 8, 10, 12, and 24 h exposure times and replaced with fresh receptor medium. The collected samples were filtered through a PTFE membrane filter (φ = 0.45 µm) and the filtrates were analysed using HPLC. There were three replicates for each experiment. After 24 h exposure, MSKE distribution was measured in the different skin layers. The stratum corneum side of the skin surface was washed with 4 × 0.5 mL water to remove the residual sample. The receptor phase was removed and the Franz diffusion cells were dismantled. The skin exposed to the sample (0.785 cm^2^) was punched out. The epidermis was separated from the dermis using the hot-plate separation method [43] at 60 °C for 2 min. Each skin layer was weighed and grinded (Mini-BeadBeater-1, BioSpec Products Inc., Bartlesville, OK, USA) into the HPLC mobile phase. Tissue suspensions were centrifuged at 14,500 rpm for 10 min, the supernatants were filtered through the membrane filter, and the filtrates were assayed using HPLC. The amount of MSKE that penetrated into the skin layers and permeated to the receptor phase was calculated from the content analysis of PGG in MSKE.

### 3.14. The Non-Perfused Pig Ear Test for Skin Irritance

The European Centre for the Validation of the Alternative Methods-recommended procedure was followed with slight modifications [35]. The pig ear skin was prepared as in the skin permeation experiment. The skin was mounted on vertical Franz-type glass diffusion cells containing 9 mL 0.01 M PBS (pH 7.4). During the experiments, the temperature was maintained at 37 ± 1 °C. The Franz cells were placed in a water bath overnight and the following morning, transepidermal water loss (TEWL) was measured using a Tewameter (MPA 5, Courage + Khazaka lectronic GmbH, Cologne, Germany). Each sample (0.5 g) studied was placed on the stratum corneum side of the skin surface in the donor compartment. Following a 4-h exposure of the pig ear to the test material, the formulations were removed and the skin surface was cleaned using 4 × 0.5 mL PBS and dried with a cotton swab. The TEWL was measured again 4 h after the formulation removal. If the mean absolute increase in TEWL was ≥6 g/m^2^/h, the tested sample was predicted to be an irritant. The SDS solution (20% w/v) and water were used as positive and negative controls, respectively. The measurements were performed in triplicate.

### 3.15. Ex Vivo Skin Corrosion Study

To determine the corrosive potential of the MSKE-loaded microemulsion formulation, an *ex vivo* method was developed that was derived from the Corrositex^®^ test [37,44]. Corrosive substances can destroy epidermis proteins and result in a colour shift in an underlying chemical detection liquid. The formulation corrosive potential was determined on pig ear skin. Nitric acid (HNO_3_, 37% v/v) and NaCl (0.9% w/v) solutions were used as positive and negative controls, respectively. The pig ear skin was prepared as in the skin permeation experiment. The skin was mounted on vertical Franz-type glass diffusion cells containing 9 mL 0.01 M PBS (pH 7.4). During the experiments, the temperature was maintained at 37 ± 1 °C. Two hundred microliters of 37% HNO_3_ solution, 0.9% NaCl solution or test sample was deposited onto the epidermis. After 15 min, the 200 μL was removed. The epidermis was washed using 4 × 0.5 mL water to remove the residual sample. One millilitre of sulforhodamine B (SRB, skin proteins labelling dye) was deposited onto the epidermis. After 15 min, the 1 mL SRB was removed. Then, the epidermis was washed using 1 mL water. The absorbance of the wash water was measured using a spectrophotometer at 565.5 nm. The corrosive factor (F) was calculated using the equation below:

(2) $$\text{F = }\frac{\text{Absorbance of sample - Absorbance of 0.9\% NaCl solution}}{\text{Absorbance of 0.9\% NaCl solution}}$$

If F ≥ 0, then the tested sample was non-corrosive. If F < 0, then the tested sample was corrosive. The measurements were performed in three times.

### 3.16. Statistical Analysis

The results were expressed as the mean ± SD and the mean ± SEM for three replicates. The statistical analysis was performed using SPSS 13.0 for Windows. Significant differences (*p* < 0.05) between the means were assessed using one-way ANOVA, followed by Tukey’s honesty significant difference test or Dunnett’s T3 test for multiple comparisons. 

## 4. Conclusions

The present study indicates that isopropyl myristate-based microemulsion systems using mixture of polyoxyethylene sorbitan monooleate and sorbitan monododecanoate (1:1, by weight) as a nonionic surfactant pair, and different types of short-chain alcohols as a cosurfactant may be promising carriers for enhancing skin penetration and delivering MSKE for topical treatments.
